# Best practices for the execution, analysis, and data storage of plant single-cell/nucleus transcriptomics

**DOI:** 10.1093/plcell/koae003

**Published:** 2024-01-17

**Authors:** Carolin Grones, Thomas Eekhout, Dongbo Shi, Manuel Neumann, Lea S Berg, Yuji Ke, Rachel Shahan, Kevin L Cox, Fabio Gomez-Cano, Hilde Nelissen, Jan U Lohmann, Stefania Giacomello, Olivier C Martin, Benjamin Cole, Jia-Wei Wang, Kerstin Kaufmann, Michael T Raissig, Gergo Palfalvi, Thomas Greb, Marc Libault, Bert De Rybel

**Affiliations:** Department of Plant Biotechnology and Bioinformatics, Ghent University, Ghent 9052, Belgium; VIB Centre for Plant Systems Biology, Ghent 9052, Belgium; Department of Plant Biotechnology and Bioinformatics, Ghent University, Ghent 9052, Belgium; VIB Centre for Plant Systems Biology, Ghent 9052, Belgium; VIB Single Cell Core Facility, Ghent 9052, Belgium; Centre for Organismal Studies, Heidelberg University, 69120 Heidelberg, Germany; Institute of Biochemistry and Biology, University of Potsdam, 14476 Potsdam, Germany; Institute of Biology, Humboldt-Universität zu Berlin, 10115 Berlin, Germany; Institute of Plant Sciences, University of Bern, 3012 Bern, Switzerland; Department of Plant Biotechnology and Bioinformatics, Ghent University, Ghent 9052, Belgium; VIB Centre for Plant Systems Biology, Ghent 9052, Belgium; Department of Biology, Duke University, Durham, NC 27708, USA; Howard Hughes Medical Institute, Duke University, Durham, NC 27708, USA; Donald Danforth Plant Science Center, St. Louis, MO 63132, USA; Department of Molecular, Cellular, and Developmental Biology, University of Michigan, Ann Arbor, MI 48109, USA; Department of Plant Biotechnology and Bioinformatics, Ghent University, Ghent 9052, Belgium; VIB Centre for Plant Systems Biology, Ghent 9052, Belgium; Centre for Organismal Studies, Heidelberg University, 69120 Heidelberg, Germany; SciLifeLab, Department of Gene Technology, KTH Royal Institute of Technology, 17165 Solna, Sweden; Universities of Paris-Saclay, Paris-Cité and Evry, CNRS, INRAE, Institute of Plant Sciences Paris-Saclay, Gif-sur-Yvette 91192, France; DOE-Joint Genome Institute, Lawrence Berkeley National Laboratory, Berkeley, CA 94720, USA; National Key Laboratory of Plant Molecular Genetics, CAS Center for Excellence in Molecular Plant Sciences (CEMPS), Institute of Plant Physiology and Ecology (SIPPE), Chinese Academy of Sciences (CAS), Shanghai 200032, China; Institute of Biology, Humboldt-Universität zu Berlin, 10115 Berlin, Germany; Institute of Plant Sciences, University of Bern, 3012 Bern, Switzerland; Department of Comparative Development and Genetics, Max Planck Institute for Plant Breeding Research, 50829 Cologne, Germany; Centre for Organismal Studies, Heidelberg University, 69120 Heidelberg, Germany; Division of Plant Science and Technology, Interdisciplinary Plant Group, College of Agriculture, Food, and Natural Resources, University of Missouri-Columbia, Columbia, MO 65201, USA; Department of Plant Biotechnology and Bioinformatics, Ghent University, Ghent 9052, Belgium; VIB Centre for Plant Systems Biology, Ghent 9052, Belgium

## Abstract

Single-cell and single-nucleus RNA-sequencing technologies capture the expression of plant genes at an unprecedented resolution. Therefore, these technologies are gaining traction in plant molecular and developmental biology for elucidating the transcriptional changes across cell types in a specific tissue or organ, upon treatments, in response to biotic and abiotic stresses, or between genotypes. Despite the rapidly accelerating use of these technologies, collective and standardized experimental and analytical procedures to support the acquisition of high-quality data sets are still missing. In this commentary, we discuss common challenges associated with the use of single-cell transcriptomics in plants and propose general guidelines to improve reproducibility, quality, comparability, and interpretation and to make the data readily available to the community in this fast-developing field of research.

## Introduction: plant-specific challenges for single-cell approaches

**Figure 1.**
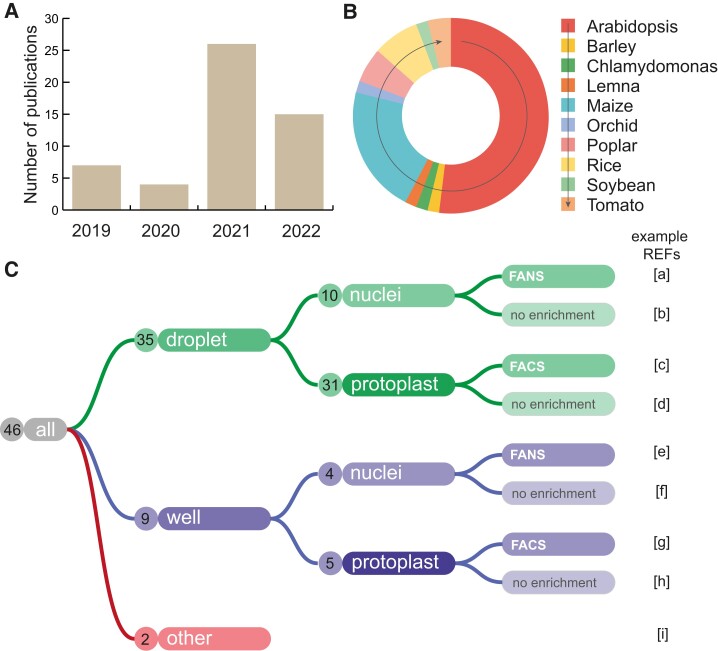
Overview of plant single-cell and single-nucleus experiments. **A)** Number of publications describing sc/snRNA-seq data in the plant field per year from 2019 until 2022. **B)** Distribution of species used in these papers (*n*: 46). **C)** Overview of the different sc/sn technologies and their usage in the plant field. Example references used: a. (Farmer et al. 2021; Cervantes-Pérez et al. 2022; Conde et al. 2022; Neumann et al. 2022; Sun et al. 2022; Guillotin et al. 2023; Li et al. 2023; Liu et al. 2023); b. (Tian et al. 2020; Marand et al. 2021); c. (Wendrich et al. 2020; Graeff et al. 2021; Lopez-Anido et al. 2021; Ortiz-Ramírez et al. 2021; Wang , Huan, et al. 2021; Apelt et al. 2022; Otero et al. 2022; Kim et al. 2023); d. (Denyer et al. 2019; Jean-Baptiste et al. 2019; Ryu et al. 2019; Shulse et al. 2019; Turco et al. 2019; Zhang et al. 2019; Satterlee et al. 2020; Bezrutczyk et al. 2021; Chen et al. 2021; Gala et al. 2021; Kim et al. 2021; Liu et al. 2021; Ma et al. 2021; Yang et al. 2021; Zhang , Chen, et al. 2021; Zhang, Chen, and Wang 2021; Li et al. 2022; Shahan et al. 2022; Tao et al. 2022); e. (Kao et al. 2021; Picard et al. 2021; Sunaga-Franze et al. 2021; Abramson et al. 2022; Li et al. 2023); f. none; g. (Efroni et al. 2016; Lopez-Anido et al. 2021; Roszak et al. 2021; Serrano-Ron et al. 2021; Omary et al. 2022); h. (Zong et al. 2022); and i. (Nelms and Walbot 2019; Song et al. 2020; Xie et al. 2022).

Plant molecular and developmental biologists are fully embracing single-cell applications. Specifically, single-cell RNA-sequencing (scRNA-seq) and single-nucleus RNA-sequencing (snRNA-seq) are gaining a lot of traction while spatial transcriptomics is emerging as a promising complementary technology (Fig. 1). Despite an increase in the use and publication of plant single-cell experimentation (Fig. 1A), it is fair to say that the plant field has, so far, not settled on common strategies, protocols, or analysis methods. Given the high complexity of the different technologies and sample types (Fig. 1, B and C), we feel it is important to provide a best-practice workflow and guidelines that will help in establishing a collectively accepted quality cutoff. These guidelines will aid in the evaluation of experimental approaches and computational analyses of single-cell transcriptomic data, while also offering solutions to commonly observed challenges, thereby improving the reproducibility and comparability of experiments in the broader field of plant research. The present coauthors collectively accept these guidelines and commit to applying them to their research. We also highlight examples where consensus has not yet been achieved between coauthors, which will need to be resolved when both the technologies and the field develop further. As one example, single-cell multiomics and spatial transcriptomics are, in our opinion, not established enough in the plant field to propose any sort of definitive rules at this moment in time.

**Figure 2.**
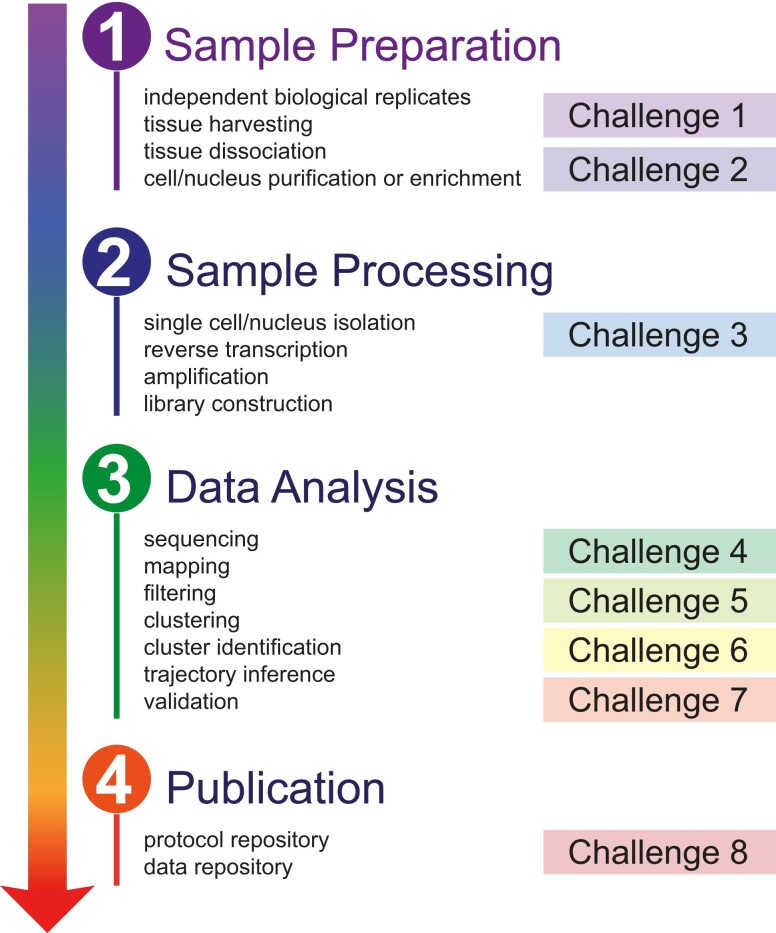
Challenges in plant single-cell and single-nucleus transcriptomics. Overview of the different steps of performing sc/snRNA-seq in plant samples and summary of how the most important challenges discussed here influence this flowchart.

To date, we have identified the following 8 main challenges in the field of plant single-cell/nucleus (sc/sn) transcriptomics: (i) deciding on the best single-cell methods to answer a specific biological question; (ii) understanding experimental variability; (iii) biases in protocols and platforms; (iv) deciding on a sequencing strategy; (v) generating expression matrices and defining high-quality cells; (vi) constructing cell clusters and mapping them to cell types; (vii) trajectory inference methods and applications; and (viii) documenting and publishing data sets (Fig. 2). Each of these challenges is discussed in detail in the following sections.

## Challenge 1: selecting the best approach to answer a specific biological question

Before considering the best experimental approach to obtain single-cell transcriptomic data, it is important to evaluate the potential benefits of accessing single-cell resolution over bulk RNA-sequencing (bulk RNA-seq). This assessment depends on the biological system considered and the biological questions to answer. sc/snRNA-seq provides a snapshot of the transcriptome of each cell within an organism, offering a high spatiotemporal resolution of the dynamic gene regulation involved in plant development, cell differentiation, or responses to biotic and abiotic stresses. Single-cell transcriptomics can also offer the required resolution to study cell-type-specific responses during cellular evolution and adaptation mechanisms among plant species (Guillotin et al. 2023). We thus advocate using sc/sn transcriptomic technologies over bulk RNA-seq when working with a cellularly complex sample or to capture dynamic transcriptomic responses to stimuli. In other cases, the question at hand might be more easily addressed using bulk RNA-seq or targeted gene expression experiments.

### scRNA-seq versus snRNA-seq

A thorough understanding of the different strategies and types of protocols (Fig. 1C) is essential before one can make an educated decision on which technology will best answer a specific biological question. A first example of an important choice is whether to profile the transcriptome from isolated nuclei or cells. When doing scRNA-seq, the most popular choice to obtain single cells from a plant organ requires the enzymatic digestion of cell walls and the generation of so-called protoplasts. There are a number of disadvantages of using protoplasts such as some tissues (e.g. sclerenchyma) and species (e.g. sorghum; Guillotin et al. 2023) are recalcitrant to cell wall digestion; enzymatic digestion affects the transcriptional status of the plant cells and could bias the outcome of experiments (Birnbaum et al. 2005); and the large size of protoplasts reduces their capture efficiency with most of the currently available commercialized single-cell platforms. Nucleus isolation followed by snRNA-seq gained traction in plant single-cell transcriptomics as well. However, the recovered data content per nucleus (e.g. Unique Molecular Identifier (UMI) or genes) is up to 10 (for UMIs) and 3 times (for genes) lower compared to scRNA-seq (Farmer et al. 2021; Guillotin et al. 2023). Furthermore, even though the transcriptome coverage is similarly efficient between scRNA-seq and snRNA-seq (e.g. 89% of all *Arabidopsis* expressed genes were captured in snRNA-seq data; Farmer et al. 2021; Guillotin et al. 2023), a nuclear transcriptome and a cellular transcriptome are not equivalent (Lee and Bailey-Serres 2019; Reynoso et al. 2019). For example, differences in abundance and composition between transcripts obtained from nuclear versus polyA RNA under hypoxia point toward nuclear transcript retention or enrichment as part of the stress response (Lee and Bailey-Serres 2019; Reynoso et al. 2019). Furthermore, the half-life of the transcripts (estimations range between 12 min and more than 24 h in *Arabidopsis* cells; Narsai et al. 2007) suggests that the cellular transcriptome is the result of the accumulation of the transcript synthesis over time, while the nuclear transcriptome is considered to accommodate faster to changes in gene activity. These differences are important to consider when selecting and later interpreting a single-cellular transcriptome versus a nuclear-based transcriptome and should be determined by the experimental system or biological question. Therefore, when studying, e.g. early stress responses of plant cells, a snRNA-seq could achieve higher resolution of rapid transcriptomic changes, while scRNA-seq might be more informative when understanding the biology of a cell type or when studying cells that are enucleated at some stages of development (e.g. sieve element cells in the phloem cell lineage; Miyashima et al. 2019).

### Biological replicates in single-cell transcriptomics

As for all scientific observations, generating robust sc/sn data sets requires performance evaluation across multiple, independent biological replicates. We hereby note that a biological replicate relies on the independent growth, harvesting and processing of various plant samples. Any separation after protoplast or nucleus isolation cannot be classified as biological replicates and can only be reported as technical replicates. No standardized metrics are available within the community to evaluate reproducibility between replicates. We propose that a correlation coefficient of the average gene expressions among all cells would be an informative assay. Alternatively, one could compare the frequency of cell types or cell clusters across replicates. As such, we advise analyzing cell cluster-specific differentially expressed genes and annotating each replicate separately, before merging the replicates and applying batch effect correction. Other parameters, e.g. Average Silhouette Width and Adjusted Rand Index, have been used to quantify cell-type purity assessments after batch effect correction and can also be informative to evaluate replicate robustness (Tran et al. 2020).

From a statistical point of view, independent biological replicates are unconditionally advised to increase the significance of biological data sets (Heumos et al. 2023). However, in many cases, replicates in sc/snRNA-seq experiments are currently performed to increase the total number of cells or nuclei analyzed, while the replicate information and a comparison between replicates are not necessarily incorporated in the actual statistical analysis. A statistical comparison among biological replicates is thus strongly advised to ensure high data quality and to prevent cluster formation based on replicate-specific artifacts. As such, biological replicates are imperative to add certainty on the reproducibility of the experiment. However, merely adding biological replicates does not remove transcriptional artifacts introduced during sample preparation in each of the replicates. One example is the effect of the enzymatic digestion needed to generate protoplasts or the procedures to extract nuclei on the transcriptome. Therefore, performing replicates by themselves does not provide sufficient confidence in the data to draw biological conclusions. To achieve confident biological interpretation, extensive downstream experimental validation is always required in the form of e.g. reporter lines, in situ hybridization, or spatial transcriptomics.

## Challenge 2: experimental variability during sample preparation

While the potential of sc/snRNA-seq for plant research is evident, its applicability depends largely on establishing reliable cell and nucleus isolation protocols. These protocols must support the generation of high-quality, high-yield nuclear and viable cellular suspensions within a short amount of time and must be compatible with downstream procedures (e.g. limited usage of PCR inhibitors like CaCl_2_). The efficiency of protoplast generation from tomato roots for example was increased by optimizing the pH of the enzyme-containing buffer and, in part, also by using hand sections instead of intact tissues (Omary et al. 2022). Preincubation in L-cysteine and sorbitol for roots of maize, sorghum, and Setaria improves enzymatic cell wall digestion and protoplast generation (Ortiz-Ramírez et al. 2018, 2021), while L-arginine positively influenced the survival rate of maize meristem protoplasts (Satterlee et al. 2020). In contrast, nuclei can be isolated from fresh (Farmer et al. 2021; Picard et al. 2021; Cervantes-Pérez et al. 2022; Conde et al. 2022; Sun et al. 2022; Liu et al. 2023), frozen (Sunaga-Franze et al. 2021; Abramson et al. 2022; Neumann et al. 2022; Li et al. 2023), or fixed (Kao et al. 2021) starting material, offering flexibility in terms of sample handling and preparation, while simultaneously securing dynamic transcriptional changes upon their rapid fixation. While nucleus isolation seems more straightforward to conduct than protoplast isolation, the assessment of nucleus quality prior to snRNA-seq library construction remains a difficult task. The leaking and clumping of isolated nuclei should be seen as a sign of breakage of the nuclear membrane leading to RNA leakage and the generation of low-quality libraries.

Overall, careful workflow optimization should include the following:

1. Visual assessment of tissue digestion or nucleus release through e.g. the observation of protoplasts/nuclei produced from all desired cell types, via cell wall digestion of fluorescently tagged cells of a particular cell type (if available) as a proxy or via gene expression quantification of cell-type markers in a pilot experiment.
2. Rapid and nondestructive sample cleanup strategies including washing steps (e.g. centrifugation and filtration), fluorescence (and image-based) activated cell/nucleus sorting (FACS/FANS), or microfluidic cell enrichment devices can increase the population of viable cells and the purity of cellular/nuclear suspensions. A careful analysis of nucleus shape will help to identify problems with RNA leakage.
3. Careful analysis of cell sizes for protoplasts since most commercial platforms have a cell size restriction that might introduce a bias in cell capturing and a preference for incorporating smaller over larger cells (see Challenge 3).
4. Quantification of cell viability by manual cell counting (upon staining with trypan blue or fluorescein diacetate) or with the help of automated cell counters.

The procedure of cell wall digestion itself (Birnbaum et al. 2005)—as well as external factors introduced during sample collection and generation (e.g. growth and harvesting conditions, enzyme concentration and activity, and temperature and timing)—affects cell viability, cell wall digestion efficiency, cell-type representation, and the transcriptional profiles of cells. One of the most promising developments for reducing experimental biases is the inclusion of a fixation step. Until now, scRNA-seq-compatible cell fixation protocols have mainly been described in mammalian research (Attar et al. 2018; Wohnhaas et al. 2019; Phan et al. 2021; Wang, Yu, and Wu 2021), but its application could drastically boost the plant single-cell field by massively reducing the effects of external factors during sample processing, including the generation of protoplasts. Indeed, protoplast isolation efficiency increased when plant tissues were fixed and digested at optimal enzyme activity temperature (Marchant et al. 2022). However, concerns about tissue fixation on protoplast shape (Marchant et al. 2022) and the sequencing results have been reported, motivated in particular by the reduction of cDNA yields and biases toward 3′-end enrichment (Wang, Yu, and Wu 2021). Despite these limitations, the potential gains for the field could be major, warranting dedicated investments in tissue fixation approaches.

## Challenge 3: biases and specificities of commercial platforms for plant single-cell transcriptomic samples

The most popular commercial platforms and scRNA-seq protocols used for plant samples rely on microfluidic droplet-based cell compartmentalization or nanowell-based cell separation (Fig. 1C). Techniques that can be performed by manual handling, such as combinatorial barcoding (Cao et al. 2017; Rosenberg et al. 2018), are rapidly expanding in the animal field but have yet to be shown in use for plant samples. The choice of the sample processing method or platform must be taken carefully to allow uniform cell size capture rate, resolvability, and, if necessary, a sample multiplexing option or flexibility toward cell capturing and lysis steps. Droplet-based platforms allow fast cell/nucleus processing but offer limited flexibility regarding the cell preparation workflow. Also, the level of pressure imposed on the sample when creating the emulsion could cause the bursting of cells into the droplet-based platform. Well-based methods like SMART-Seq2 (Lopez-Anido et al. 2021) and platforms used with plant samples, such as BD Rhapsody (Zong et al. 2022) or iCELL8 (Sunaga-Franze et al. 2021), require longer cell processing protocols but offer more flexibility during the sample processing. However, the compatibility of commercial platforms to handle the size and fragility of plant protoplasts is not necessarily evaluated. Plant cell sizes typically lie in the range of 10 to 80 *µ*m, with even larger values observed for endoreduplicated cells, which is far above the recommended cell size maxima from current technology providers (∼40 *µ*m). Furthermore, cell size heterogeneity can create cell capture biases, because droplet-based techniques favor smaller cells, while the well sizes in nanowell techniques must be fine-tuned to reduce the possibilities of doublets from smaller cells while still allowing capture and processing of larger cells. The consequences could be high multiplet rates and/or imbalanced cell-type/stage representation. Careful optimization of the maximum cell loading capacity, loading speed, cell compartmentalization time, and the number of washing steps is necessary depending on the platform of choice. Identification of cell type or stage capture rates, however, requires in vivo experimental validation by quantification of cell types or tracing of developmental cell stages. This validation has been done by comparing cell numbers per cell type between scRNA-seq data and cell counting via imaging (Wendrich et al. 2020), but it could also be achieved by spiking in a fixed ratio of cell types using transgenic marker lines.

Furthermore, a detailed plant-specific benchmark study comparing the commercially available platforms and kits is urgently needed to evaluate the benefits and pitfalls when applied to plant samples. Similar benchmark studies using human and mouse cell lines allowed practicality and financial comparisons of common methods, while also comparing cell capture rates and technical bias across cells with distinct cell properties (Mereu et al. 2020). Ideally, a benchmark study should cover numerous species and tissue combinations to allow the establishment of quality standards independent of the species or tissue used. A plant-specific benchmark study might in addition also focus on comparing the ability to recreate developmental or spatial cell states, because most plant single-cell samples offer a chance to also capture and compare developmental cell trajectories. Given the sensitive nature of protoplasts, it would also be important to address the effect of total sample processing durations, as well as the abundance of ambient RNA due to for example protoplast bursting.

## Challenge 4: establishing an efficient sequencing strategy

### Full-length versus 3′- or 5′-end transcript sequencing

Two approaches for library preparation are currently used in single-cell methodology, namely full-length transcript coverage and 3′- or 5′-end transcript coverage. While most reported plant single-cell transcriptomic data sets today (Fig. 1C) were generated using 3′end transcript coverage, full-length transcript sequencing (used for example in Lopez-Anido et al. 2021) bares huge application potential in plant research, as it can help in improving transcriptome and epitranscriptome resources (reviewed in Shen et al. 2023) and in providing cell-type-selective isoform usage (shown for example during neuronal maturation in mouse embryos; Lebrigand et al. 2020).

### Cell number and sequencing depth

Although cDNA amount and profile after library preparation are used as a proxy for the overall quality of the sc/snRNA-seq library, sequencing followed by analysis currently remains the only way to fully estimate its quality and biological value. Two major issues that require careful consideration during the experimental planning and sample optimization are the number of cells/nuclei needed for optimal coverage of the cell type(s) of interest and the aimed sequencing depth per cell/nucleus. The number of cells/nuclei in published cell atlases is moving from thousands to hundreds of thousands. Increasing numbers is indeed beneficial for predicting novel marker genes, because it allows better coverage of rare populations and lowly expressed genes. This higher coverage in turn helps to outweigh the bias of differential expression analysis tools toward highly expressed genes (Squair et al. 2021) and data set-specific noise (Fischer and Gillis 2021). However, at what point does a cell atlas fully capture the cellular diversity of its samples? The meristematic region of an *Arabidopsis* root has about 3,000 to 4,000 cells, meaning that a data set of 100,000 cells sufficiently covers each cell about 20 to 30 times, assuming that all cells are equally represented in the data set. Alternatively, when a specific cell type is isolated from a tissue by upstream cell sorting, an atlas of 2,000 cells could already be saturating. As a rule of thumb, when the cell-type composition of the tissue of interest is known, the minimum number of cells that need to be analyzed can be estimated from the probability to robustly capture the rarest cell type(s) (e.g. https://satijalab.org/howmanycells/). For example, to obtain at least 10 quiescent center cells (estimated to represent 0.1% of all cells within the *Arabidopsis* root meristem according to Cartwright et al. 2009; Shahan et al. 2022) with 95% confidence, one would need to profile 15K to 20K cells.

Additionally, the required sequencing depth must be adapted depending on the biological question, the tissue complexity, and the sample quality. However, it is recommended that optimal coverage is given with 1 read/cell/gene (Zhang et al. 2020). Alternatively, sequencing can be staged by first initiating a shallow sequencing of the library (e.g. up to 10,000 reads/cell) before deeper sequencing (e.g. aiming for 50,000 reads/cell). Such shallow sequencing allows evaluating the performance of cell cluster analysis and annotation and is sufficient to capture the entire cell-type heterogeneity of the sample (Zhang et al. 2020). Another common suggestion for a preliminary sample quality control is to analyze the expression of a gene subset related to a biological question (Zhang et al. 2020). Sequencing even less for testing the quality of the library is possible but will affect the retrieval of cell types with a lower number of transcripts, which might be lost within the background of empty droplets if the sequencing is too shallow. The desired final read depth will depend on the goal of the experiment but should ensure sequencing enough cells at a sequencing depth that captures individual events robustly (e.g. 50% sequencing saturation; Table 1).

**Table 1.** Necessary reported information to allow evaluation and repetition of a plant sc/sn experiment

	Details	Recommendations
**Biological material**	Species	e.g. *Arabidopsis thaliana*, *Zea mays*
	Accession	e.g. Col-0
	Genotype	e.g. WT or mutant background
	Tissue type	e.g. root, leaf, stem, and seed
	Detailed growth conditions	e.g. temperature, light conditions, and medium
	Harvest conditions	e.g. age of plants, time of day, and amount harvested
**Sample preparation**	Isolation protocol	Short description of the way the sample was isolated
	Tissue dissection	e.g. razor blades, needles, and tissue homogenizer
	Fixation	Short description of the way the sample was fixed if this was done
	Cell/nucleus enrichment	e.g. sucrose gradient, FACS (incl. model, nozzle size, and temperature)
	Total sample preparation time	For cells: <90 min for *Arabidopsis* roots (from material harvest to cell loading)* *Duration may increase depending on starting material and time needed for optimal tissue digestion For nuclei: 30 to 60 min (depending whether a nucleus enrichment step is included)
	Estimated cell/nucleus number loaded	An estimation of the amount of cells or nuclei loaded, based on the cell/nucleus concentration and volume that was loaded
	Instrument/method/kit	e.g. 10× Genomics 3′ v3.1, BD Rhapsody WTA
	Cell viability test	For cells: trypan blue, fluorescein diacetate, calcein, propidium iodide, 4′,6-diamidino-2-phenylindol For nuclei: not applicable
**Libraries**	Library construction	Protocol and revision/version that was followed, e.g. CG000204 Rev D for 3′ v3.1
	Amplification method	e.g. number of PCR cycles used for cDNA amplification
	End bias	e.g. 3′-end mainly; excess of rRNA or TSO sequences
**Sequence results**	Instrument/method	e.g. NovaSeq, NextSeq, ONT, and DNBSEQ
	Library layout/paired-end	Consider to use standardized library structures (Booeshaghi et al. 2023)
	N° sequenced reads	20,000 to 50,000/cell for RNA or more 20,000 to 40,000/nucleus for RNA or more
**Raw data**	Reference genome	Link to Ensembl Plant fasta file, JGI, NCBI, PLAZA
	Annotation version	If custom annotation, also include.gtf/.gff/.gff3 files
	Mapping method (incl. software, customized settings)	e.g. STAR (cellranger)
	Mapping efficiency	>85% for Arabidopsis* *Value may be lower in other species
	Sequencing saturation	>50%
	Estimation of ambient RNA	Fraction of reads in cells > 60% for scRNA-seq Fraction of reads in cells > 50% for snRNA-seq
	Imputation method and settings	If relevant
**Processed data**	N° captured cells/nuclei	e.g. 60% of estimated number
	N° high quality cells/nuclei	e.g. 20% of estimated number
	Filter criteria: % mitochondrial reads/cell or nucleus	<10% for scRNA-seq* close to 0% for snRNA-seq* *value may deviate depending on biological context
	Filter criteria: % chloroplast reads/cell or nucleus	<5% to 10% for scRNA-seq* close to 0% for snRNA-seq* *values may be higher depending on biological context
	Filter criteria: Minimum N° UMI/cell or nucleus	>1000 for scRNA-seq>400 for snRNA-seq
	N° total detected transcripts	Dependent on the species but e.g. 60% of total number of transcripts in the annotated genome
	Doublet rate	Estimates according to 10× Genomics user guide based on number of loaded cells/nuclei
	Replicate comparisons	Provide coefficient correlation of the most variable genes or cluster-specific genes between independent replicates or compare pseudo-bulk from sc/snRNA-seq versus bulk RNA-seq
	Batch correction method for merging (incl. reasoning for batch correction)	e.g. Seurat, Harmony
	Additional processing	e.g. removal of protoplast-induced genes, cell cycle regression, noise (ambient RNA) removal, cluster membership bias between replicates, removal of low quality clusters
**Validation**	Method of automatic annotation of clusters	e.g. Label transfer, spatial transcriptomics
	Method of manual annotation (markers, gene function info)	e.g. Marker genes, orthologous, correlation with bulk RNA-seq and microarray data; Index of Cell Identity calculation
	Verification in planta (e.g. number of markers used for validation)	e.g. Spatial transcriptomics; RNA in situ hybridization; promoter fusions
**Data availability**	Analysis scripts & codes (GitHub)	If relevant
	Excel Tables DEG for each cluster	e.g. Lists for each cell type/developmental stage from FindMarkers (Seurat)
	Objects/count matrix in repository (which one, where?)	e.g. use NCBI GEO to store count matrices and Seurat object
	On-line tool/browser URL	List the URL if relevant
	Cell-level metadata table	Include cell-type annotations for each cell barcode
**Additional**	additional comments from the authors	e.g. annotation/counting of rRNA, allow for rRNA estimation, consideration of intronic reads

A downloadable empty version for use in publications can be found as Supplementary Table S1. If numerical values deviate from the recommended numbers provided below, an explanation should be provided. The numerical values in this table are derived from available studies in plants, most of which originated from *Arabidopsis*.

When assessing the most cost-effective sc/snRNA-seq technologies, should one profile many cells/nuclei but have shallow sequencing or should one profile fewer cells/nuclei but with deeper sequencing? In many cases, the ideal scenario will be something in-between assuming that the researchers are working on high-quality cells/nuclei. Nevertheless, a choice toward either a higher number of cells/nuclei or higher sequencing depth can be made depending on the biological question, the quality of the biological entities used for the analysis, and the relative abundance of each cell type composing the organ. If the aim is to generate an atlas potentially uncovering rare cell types, a better strategy would be to profile many cells/nuclei with a lower sequencing saturation. However, a minimal depth in sequencing (Table 1) upon maximizing cell/nucleus quality is still necessary to ensure that low-abundance transcripts that define rare cell types are captured and to saturate the transcriptome of the sample. Validation of high-throughput technologies in the plant field that enable to access the transcriptomes of hundreds of thousands and even up to 1 million cells or nuclei, combined with the ongoing expansion of sequencing capabilities and the decrease in sequencing costs, could help to overcome this dilemma. If the goal is to do functional gene discovery and generate gene regulatory networks for example (Ferrari et al. 2022), a high sequencing saturation per cell/nucleus favors the discovery of low-abundance gene transcripts. To achieve this goal, one can select a subset of genes related to the biological question and adjust the sequencing depth until at least 1 read/cell for each of those genes is reached (Zhang et al. 2020). In all cases, we recommend optimizing cell and nucleus isolation methods to ensure the capture of the largest number of transcripts from each biological entity.

## Challenge 5: generating an expression matrix from high-quality cells/nuclei

**Figure 3.**
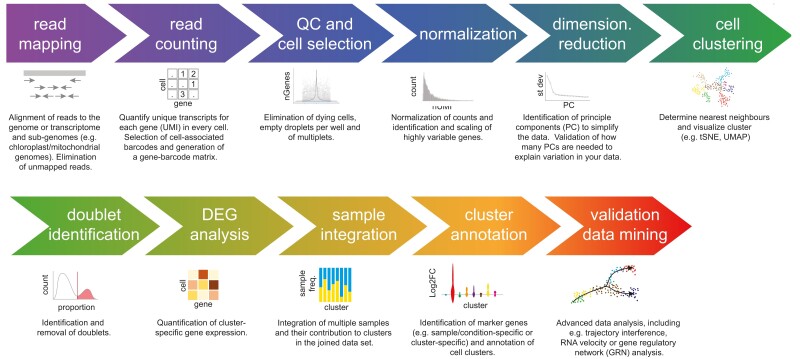
Workflow for sc/snRNA-seq data analysis.

Once mRNA sequence reads are obtained, reads are mapped to the genome and ultimately to genes and cells of origin using a reference genome and UMI and cellular barcode information. Standard data analysis workflows further include quality control filtering, quantification of gene expression in each cell, clustering, and visualization of cells based on transcriptomic similarity (Fig. 3). While the recommendations below are given with the intention to standardize sample quality parameters within the field (see recommendations in Table 1), we want to highlight that a sn/scRNA-seq experiment (with a read depth of 30K reads/nucleus or cell) should typically result in the capture of 1,000 expressed genes/nucleus or 3,000 expressed genes/cell, respectively. Furthermore, utilizing universal preprocessing pipelines for single-cell genomic data (e.g. Booeshaghi et al. 2023) can help in streamlining cell quality filtering and enhance data reproducibility in the future.

### Read mapping

Plant genomes in general and crop genomes in particular are poorly annotated compared to e.g. human or mouse genomes. Moreover, due to frequent whole-genome duplications (Fox et al. 2020), many plants are polyploid and thus contain multiple similar copies of each gene. Structural annotations of genes are especially important for most droplet-based technologies using a 3′ capturing strategy to properly map the sequencing reads and to quantify transcript abundance. The distribution of the mapped reads on the genome can give an indication about the quality of the annotations. Poor mapping efficiency consequently causes gene loss, which can be dramatic especially for popular 3′-based single-cell technologies. Even for *Arabidopsis* scRNA-seq data sets, mapping rates vary but should be e.g. >85% (Table 1). A high percentage of reads mapping to intergenic regions (e.g. >20%) can be an indication either that not all genes are annotated or that the annotated 3′ UTR regions should be longer. As a note, it is important to mention that the sequencing reads generated upon conducting a snRNA-seq experiment should be mapped against the exonic and intronic sequences of the annotated transcripts, a reflection of the capture of spliced and unspliced transcripts.

### Removal of low-quality cells/nuclei

**Figure 4.**
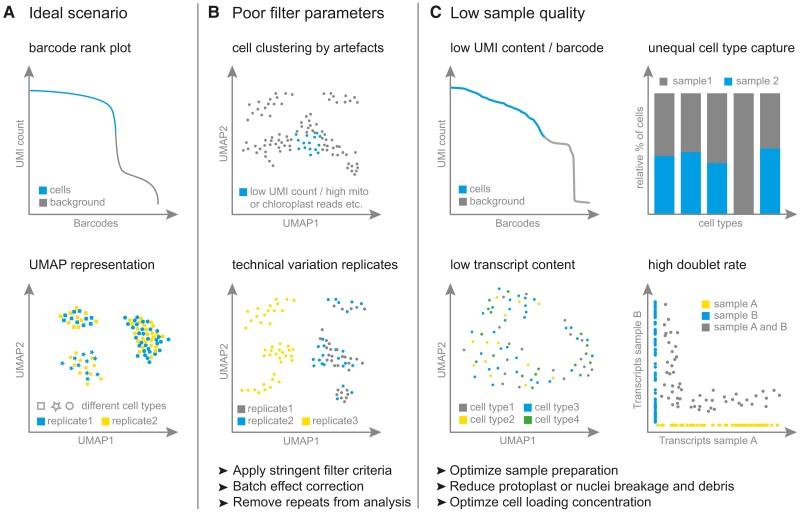
Overview of common problems in sc/snRNA-seq analysis and possible solutions.

After read mapping, low-quality cells/nuclei (e.g. cells/nuclei with low number of UMIs and genes) need to be filtered out (see numerical recommendations in Table 1). Impairments in applying low-quality cell filtering may increase the noise in the data set and reduce the accuracy in downstream analysis, including cell clustering or erroneous identification of cell types (Fig. 4, A and B). General filter parameters will depend on the application and sample type (i.e. cells versus nuclei). For plants, it is good practice to exclude cells with high mitochondrial (e.g. >10%) and chloroplast (e.g. >5% to 10%) reads (Table 1). Such cases may indicate cells under stress because of perturbations during the sample preparation. These values might need to be adapted when studying highly or lowly metabolically active cells or cells undergoing e.g. programmed cell death. If no mitochondria or chloroplast genome is available, plotting the number of genes versus the total UMI count can be used instead to show cells with low data content. The barcode-rank-plot (“knee plot”) is a commonly used tool to determine sample quality by ranking all barcodes according to their UMI content (Fig. 4A). A sample of low quality can be identified if there is no clearly defined boundary between barcodes with high UMI content and barcodes with significantly lower UMI content (Fig. 4C). Similarly, poor cluster separation in a sample that fails to differentiate cell types indicates an insufficient amount of transcript content per cell/nuclei.

Ambient RNA and the presence of empty droplets and doublets—both associated with droplet and nanowell-based technologies—can also lead to noise in the expression matrices, inaccurate cell clusters, and falsely differentially expressed genes. Therefore, it is important to ensure low amount of ambient RNA introduced during sample preparation (e.g. by mixing cells/nuclei from multiple species) and to optimize cell loading concentration (Fig. 4C). Bioinformatic tools (e.g. SoupX, Young and Behjati 2020; or CellBender, Fleming et al. 2023) can be used to computationally remove transcriptional noise introduced by ambient RNA. Experimentally, even though not yet shown suitable for plant single-cell transcriptomics, doublets can be detected using antibody (cell hashing; Stoeckius et al. 2018) or lipid-tagged indices (MULTI-Seq; McGinnis et al. 2019) sample multiplexing strategies. Here, doublets are identified if the cell-specific barcode is connected to multiple antibody or lipid-tagged indices, respectively. Mixing cells/nuclei from multiple species in 1 sample offers another experimental setup to identify doublets (Shulse et al. 2019; Fig. 4C). In this case, the doublet is identified due to the cell-specific barcode being linked to multiple species. While an experimental setup that allows doublet identification would be the best practice for identifying doublets, it will not help in removing doublets from already existing data sets. Even though significant advances have been made in identifying doublets computationally (see benchmark study; Xi and Li 2021), it remains a major challenge in general and even more in plant sc/sn transcriptomic analysis due to the presence of endoreduplication. Such polyploid cells will appear as outliers when plotting the gene content or UMI/cell, but additional expression quantification of ploidy marker genes (if available) allows to distinguish endoreduplicating cells (Wendrich et al. 2020).

### Identification of protoplast-induced genes

In order to exclude that the invasive enzymatic treatments needed to generate protoplasts might influence the observed transcriptional status of certain cells or cell populations, the overall transcriptomic responses induced during these procedures should be determined using bulk RNA-seq in each experimental setup (Birnbaum et al. 2005; Brady et al. 2007). Indeed, protoplast isolation adds a definite stressing factor to each cell type (Denyer et al. 2019; Xu et al. 2021; Wang, Huan, et al. 2021). Although the absence or presence of protoplast-induced genes did not alter cell clustering or annotation in *Arabidopsis* root scRNA-seq data (Denyer et al. 2019), these genes should at least be flagged in the data set to avoid misinterpretation.

## Challenge 6: cell cluster identification and annotation

### Data normalization, dimensionality reduction, and cell cluster visualization

Considering the limited number of transcripts per cell, single-cell transcriptomics fail to detect transcripts for most genes in a given cell. This sparsity is further enhanced by intrinsic noise from stochastic transcript fluctuations, cell-cycle state, and cell heterogeneity among other biological factors. As a result, it is necessary to implement scRNA-seq-specific normalization and batch correction protocols (see Luecken and Theis 2019 for a review on this specifically). Clusters of cells (i.e. cells that share similar expression profiles) are constructed using community detection algorithms, which control the degree to which similar cells should be grouped together or stay separate based on preset parameters. To visualize the data, “dimensional reduction” algorithms are applied to the data, typically via Principle Component Analysis (PCA), t-distributed Stochastic Neighbor Embedding (t-SNE), or Uniform Manifold Approximation and Projection (UMAP). However, a word of caution is necessary here: whereas PCA involves linear projections, t-SNE and UMAP are nonlinear transformations introducing significant distortions (Chari and Pachter 2023). Indeed, visualizing a synthetic data set with tSNE or UMAP revealed that cluster distances and locations in discrete and trajectory simulations are inaccurately represented compared to the defined distances in the original data (Wu et al. 2018). These results indicate that cluster distances or locations cannot be used alone to draw biological conclusions. If different clustering outcomes (produced with customized parameters) seem equally plausible, it is appropriate to apply the next step—mapping clusters to cell types—to each possibility and then use that extra information to decide which clustering makes most sense from a biological point of view.

### Cell cluster annotation

**Figure 5.**
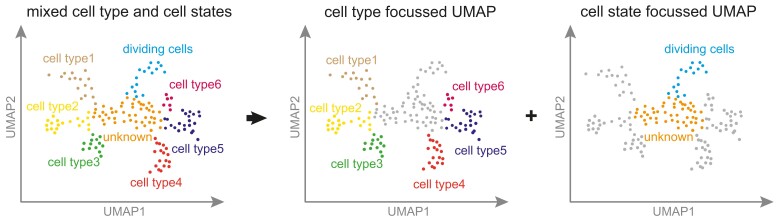
Cluster annotation and appropriate labels. A common technique to represent single-cell RNA-seq data requires mapping of each cell’s transcriptome to a low (typically 2-) dimensional domain (e.g. tSNE or UMAP), after which highly similar cells group together into initially unannotated clusters. Annotating cells to a cell type or developmental stage is important for further interpretation of transcriptomic signatures. Traditional, manual annotation methods screen differentially expressed genes within each cluster for the presence of individual marker genes or by transferring knowledge on cluster annotation from a reference data set to an unannotated data set (reviewed in Clarke et al. 2021). Without prior knowledge of marker genes and reference data sets, automated annotation methods (e.g. eager, lazy, and marker learning methods; Xie et al. 2021) can aid in assigning labels based on comparing cell cluster-specific genes and their biological functions. Cells and clusters that cannot be annotated with high confidence to (only) one cell type must be analyzed carefully to determine if they represent a mix of subcell types and/or cell states. Although there is currently no standardized definition of a “cell type” or a “cell state” (discussed in more detail in Amini et al. 2023), it has been proposed that a cluster with homogenous marker gene expression among all cells likely represents a cell type, while expression gradients among the cells within a cluster represent cell states (Clarke et al. 2021). However, as these definitions are still evolving, caution should be taken to not mix different anatomical levels (cell types, tissues, or organs) and cellular processes (e.g. cell division and cell cycle) within the same visual representation. To avoid confusion, we recommend using multiple figures with different levels of labels (e.g. separate cell types, cell division states, tissues, and so on).

The annotation of clusters (meaning mapping each cluster to a cell type or state) is facilitated using manual or automated cell annotation methods (Fig. 5). Manual annotation requires previous transcriptome knowledge gained from as many different cell types as possible. Cell-type marker genes for plant tissues in well-studied species can come from manually curated lists that are based on bulk RNA-seq data from purified cell populations (Jin et al. 2022) or from already annotated single-cell data sets. However, to date, databases containing this information are rare and restricted to a few plant species and tissues (e.g. Jin et al. 2022). One can appeal to interspecies correspondences to support the functional annotations of the clusters, assuming that a substantial fraction of orthologous genes share similar cell-type transcriptional specificities. For instance, if an atlas for another species is available, one can attempt to extend the orthology between genes to orthology between cell types. Such an approach has been very successful, leading to atlases for instance in rice (Wang, Huan, et al. 2021; Li et al. 2023), *Medicago truncatula* (Cervantes-Pérez et al. 2022), maize, sorghum, and Setaria (Guillotin et al. 2023). However, this approach also has its limitations: genes often exist in multigene families rendering the orthology mapping ambiguous, and the conservation of marker gene/cell type pairs is far from perfect (Movahedi et al. 2012). An additional limitation of these approaches is that they require multiple marker genes per cluster to ensure proper annotation.

Another option is so-called label transfer: transferring cluster labels between existing single-cell expression atlases (e.g. for roots: https://rootcellatlas.org/, or other organs: http://neomorph.salk.edu:9000/) to unlabeled data sets. Common tools used are scmap (Kiselev et al. 2018), SingleCellNet (Tan and Cahan 2019), SingleR (Aran et al. 2019), and Seurat (Stuart et al. 2019), which differ in their accuracy and their ability to handle sample- and protocol-related nested batch effect removal and the presence or absence of cell types in the reference atlas or target data set (Luecken et al. 2022). The advantage of this approach is that already published data can be directly reused. With both automated annotation approaches, it is important to consult several tools and select one final annotation, for instance using a majority rule.

As an alternative or complementary approach, clusters can be annotated manually. To achieve this, marker gene expression of cells can be visualized in a UMAP plot or via a dot plot showing cluster-specific expression of marker genes. Cell clusters with conflicting annotations or no annotation, due to e.g. low quality of the transcriptomic information or capture of uncharacterized cell (sub)type or cell transition state, should be marked as “unknown.”

### Refining cell cluster annotation

Ideally, each cluster will be clearly associated with one cell type, using any of the approaches described above. However, the current literature often mixes different levels of anatomical annotations. The ambiguity between classical anatomical descriptions and the new molecular characterizations of these cell types, tissues, and structures make it even more challenging to navigate among these definitions. Hence, to resolve these ambiguities, multiple hierarchically structured annotations can be used in which cells/clusters can be annotated according to e.g. broad expression domains, the tissue, or the cell type level (Michielsen et al. 2021). Until there is a consensus in the community, we recommend to separate the different levels of annotations (cell type, tissue, and cell cycle) into distinct plots. This will avoid ambiguity and misinterpretation of the data (Fig. 5).

### Validation of cell cluster annotation

Independent of the annotation approach used, we strongly recommend experimentally validating the main annotations by e.g. generating corresponding reporters, performing RNA in situ hybridizations, or complementing the data with spatial transcriptomics. This independent experimental approach is the only way to assess if differential gene expression observed in a high-throughput single-cell experiment is relevant in vivo or an artifact introduced by one of the many steps, e.g. protoplasting.

Beyond these more classical approaches, spatial transcriptomics can be used to support the annotation of clusters identified in plant sc/snRNA-seq data sets (Guillotin et al. 2023; Lee et al. 2023; Nobori et al. 2023). The technologies that allow probing tissue gene expression and simultaneously retaining its spatial location can be divided into two main categories: targeted and untargeted. The division into those two categories is based on the type of approach applied to analyze the tissue gene expression information. Specifically, targeted methods, i.e. in situ sequencing (Ke et al. 2013; Laureyns et al. 2021), MERFISH (Moffitt et al. 2016; Lee et al. 2023; Nobori et al. 2023), Xenium (Ke et al. 2013; Lee et al. 2015; Janesick et al. 2022; Liu et al. 2022), NanoString CosMx (He et al. 2022), Molecular Cartography (Groiss et al. 2021; Guillotin et al. 2023; Yang et al. 2023) to list a few, require a priori knowledge on which genes to study, since these approaches use gene-specific probes to fluorescently visualize and count the gene transcripts of interest in the tissue. In contrast, untargeted methods, e.g. Visium (Giacomello et al. 2017; Liu et al. 2022, Liu et al. 2023; Peirats-Llobet et al. 2023), DBiT-Seq (Liu et al. 2020), Slide-seq v2/Curio (Stickels et al. 2021; Lee et al. 2023), and Stereo-seq (Xia et al. 2022), leverage a localized capture of polyadenylated transcripts, thus allowing to obtain 2D whole transcriptomic maps. Both targeted and untargeted approaches can aid in determining the spatial location of specific cell types or stages of interest (e.g. indeterminate and determinate SAM cells in maize, defined by their expression levels of *PLASTOCHRON1*; Laureyns et al. 2021), annotate cluster identities obtained in sc/snRNA-seq experiments, and/or validate marker genes (Guillotin et al. 2023; Lee et al. 2023; Nobori et al. 2023). As such, spatial transcriptomics and sc/snRNA-seq are complementary technologies for obtaining high-resolution spatiotemporal expression data.

## Challenge 7: application of trajectory inference in plant single-cell transcriptomics

Trajectory inference can predict developmental or stress–response trajectories in sc/snRNA-seq data sets, allowing one to pinpoint e.g. cell cycle transitions or bifurcations when new specific cell identities branch off from another lineage. While trajectory interference is a very promising tool to identify novel biological phenomena and questions, it is important to understand that the pseudotemporal ordering of cells along a trajectory is purely based on transcriptomic similarities, meaning that a sufficient, unbiased sampling of cells is required, as well as prior knowledge to assign a developmental direction (see Tritschler et al. 2019 for general recommendations). Furthermore, no conclusions about the spatial organization can be drawn, and it is advised to confirm and complement the trajectory output with other methods. Multiple trajectory interference methodologies have been developed, benchmarked, and used in the animal field (Saelens et al. 2019), but they show special promise in plants because plants have many continuous developmental programs and plant cells have remarkable capabilities for dedifferentiation and adaptation. Trajectory analysis gave significant insight into recreating cell (type)-specific transcriptional events during plant developmental processes, such as lateral root development (Serrano-Ron et al. 2021), stomata development (Kim et al. 2023), root hair (Denyer et al. 2019; Shulse et al. 2019), pistil (Li et al. 2023), or phloem development (Roszak et al. 2021; Otero et al. 2022). For example, when combined with live imaging, trajectory-predicted expression gradients and cell-type-specific transcriptional networks allowed a complete reconstruction of the developmental process and a precise, cell-by-cell lineage tracing during protophloem development (Roszak et al. 2021). An interesting computational analysis that can be applied to verify predicted trajectories in plant tissues is making use of the ploidy increase in plant cells as they mature from meristematic cells with 2C/4C content into a more differentiated stage, marked by 8C/16C or higher content. In *Arabidopsis* root tissues, these ploidy states have been linked to specific markers, leading to predictions of the ploidy status of each individual cell. This increase in ploidy level allows to pinpoint or validate the more meristematic cells with low ploidy levels and the more differentiated cells in a sc/snRNA-seq data set with higher ploidy levels (Bhosale et al. 2018; Wendrich et al. 2020; Shahan et al. 2022).

## Challenge 8: documentation and publication of plant single-cell data sets

Recent advances in single-cell omic technologies in plants have enabled insights into diverse aspects of physiology and development and have been the centerpiece of a growing number of elegant studies. However, there is additional potential for single-cell resources through their reuse in integrative meta-analyses (showcased e.g. in Leote et al. 2022). The multiple applications would increase their power and depth through greater numbers of cells, a more comprehensive assessment of biological variation, and enhanced enrichment for different cell types or states that are targeted by individual studies. Popular methods enabled within software packages (e.g. Seurat, Monocle, Scanpy, and Harmony) (Wolf et al. 2018; Korsunsky et al. 2019; Van den Berge et al. 2020; Hao et al. 2021) have streamlined the process of stitching data sets together across samples, studies, and experimental platforms, even across tissues and species, allowing for a more expansive use of single-cell data for describing and understanding biological organization across scales.

To make optimal use of the generated data in the plant field, there is an urgent need for published single-cell data and their associated metadata to be more easily accessible and usable. First steps toward establishing a suitable infrastructure that allows data storage and comparison have been taken within the framework of the Plant Cell Atlas (Fahlgren et al. 2023). To a large extent, this FAIR (Findable, Accessible, Interoperable, Reproducible) principle has not been an issue for raw single-cell data. Unprocessed FASTQ files are deposited routinely through major data portals, such as the National Center for Biotechnology Information Short Read Archive (NCBI SRA; http://www.ncbi.nlm.nih.gov/sra), and accessibility to raw data is usually mandated by journals, funding agencies, and institutions. In most cases, well-indexed raw data are available and cited in journals. However, care must be taken for single-cell data generated through popular platforms in that only reads from the paired-end sequencing strategy, as well as indexed and/or UMI FASTQ, must be deposited. These genomic reads alone are insufficient for reconstructing the single-cell count matrix necessary for nearly all analysis steps. Processed data should also be stored in publicly accessible repositories such as the NCBI Gene Expression Omnibus (GEO; http://www.ncbi.nlm.nih.gov/geo). Processed data not only include the cell/gene counts matrix at minimum but can also include more complex data objects such as those generated by the Seurat (R) (Hao et al. 2021) or AnnData (Python) (Wolf et al. 2018) packages. The EBI Single-cell Expression Atlas is another repository that will accept processed count matrices and allows for the deposition of cell metadata (see below).

Less prevalent is the public accessibility of metadata associated with single-cell studies. This metadata takes several forms: (i) experimental metadata describing how samples were treated, what tissues were harvested, how the samples were processed, and what platforms/versions single-cell sequencing was performed on them; and (ii) imputed metadata describing attributes of cells defined by downstream analysis of the raw data, including the cell's identifier (usually a sequence barcode), the number of distinct genes detected, total transcripts detected, assigned cell type or cluster number, or other features that are described in a study but not immediately available from the raw data. Experimental metadata, including protocols used to generate samples, should be well documented in the manuscript that presents them, and we encourage depositing the protocol in a public repository. Protocols.io is emerging as a standard for this (see e.g. the repository for the Plant Cell Atlas; https://www.protocols.io/workspaces/plant-cell-atlas), and detailed sample information should go along with the raw data when submitting to a repository like GEO/SRA. Imputed metadata can be provided along with the raw data in the NCBI GEO as a simple machine-readable table (.csv or .tsv). All forms of metadata are vitally important for integrative analysis, as it is difficult, if not impossible, to recreate exactly the analysis steps performed from a published study to replicate results. In Table 1, we have listed important metadata parameters that should be accompanying all publications using sc/snRNA-seq data to ensure transparency in data quality and will allow for a better interpretation of the results and their use in larger meta-analyses. Although it is challenging to give an exact number for these parameters due to the vast differences in experimental systems, species, tissues, and technologies, we do attempt to provide a range based on the collective author's experience in processing and analyzing multiple species and organs to guide the less experienced user. An empty version of this table can be downloaded as Supplementary Table S1 and freely used for publication. All coauthors collectively commit to start using this table in all publications.

Finally, analysis scripts, software environments, and (ideally) visualization portals should be made publicly available on established repositories/portals. Where possible, a well-documented code can be stored in dedicated repositories. Analysis environments should at minimum be well documented (with versions specified for all software packages used for analysis).

Many of these tools are only valid for single-cell transcriptomic or chromatin accessibility methods that use DNA sequence as an output and are thus incompatible with other data modalities such as proteomics or metabolomics. While these data types are outside the scope of this review, they too should adhere to FAIR principles (Wilkinson et al. 2016) to ensure that they can be integrated with other data sets when the computational infrastructure develops. These data management tools are also not guaranteed to be future proof. New technologies may arise that will render existing data sets/tools obsolete. Thus, it is imperative that, as the field evolves, we do not forget about legacy data and ensure that it is preserved in a way that will be useful before this becomes impossible.

## Conclusion and outlook

Even for more advanced users, it is challenging to keep up with the rapidly evolving field of sc/snRNA-seq. Continuous advancements are not limited to the actual technology and also include the choice of which technology to use for a specific problem, which method to use to isolate high-quality cells or nuclei, and how to analyze, compare, and store these vast amounts of data. In this commentary, we discuss recommendations regarding data generation, analysis, storage, and documentation to ensure transparency in publication and optimal use of the generated data across experiments, tissues, species, and laboratories. We collectively commit to following the guidelines and recommendations (Table 1). Future research and method maturation will allow us to fine-tune and expand these guidelines and recommendations.

## Supplementary Material

koae003_Supplementary_Data

## Supplemental data

**Supplementary Table S1.** Necessary reported information to allow evaluation and repetition of a plant single cell/nucleus experiment.

## Dive Curated Terms

The following phenotypic, genotypic, and functional terms are of significance to the work described in this paper:

cluster CHEBI: CHEBI:33731
