# Supplementary material for: Best practices for the execution, analysis, and data storage of plant single-cell/nucleus transcriptomics
Source: Plant Cell. 2024 Jan 17;36(4):812–28. doi: 10.1093/plcell/koae003 (PMC10980355; doi:10.1093/plcell/koae003)
Supplement: koae003_Supplementary_Data [file koae003_supplementary_data.docx]

**Table S1: Necessary reported information to allow evaluation and repetition of a plant single cell/nucleus experiment**

|  | **Details** | **Experimental information** |
| --- | --- | --- |
| **Biological material** | Species |  |
|  | Accession |  |
|  | Genotype |  |
|  | Tissue type |  |
|  | Detailed growth conditions |  |
|  | Harvest conditions |  |
| **Sample preparation** | Isolation protocol |  |
|  | Tissue dissection |  |
|  | Fixation |  |
|  | Cell/nuclei enrichment |  |
|  | Total sample preparation time |  |
|  | Estimated cell/nuclei number loaded |  |
|  | Instrument/Method/Kit |  |
|  | Cell viability test |  |
| **Libraries** | Library construction |  |
|  | Amplification method |  |
|  | End bias |  |
| **Sequence results** | Instrument/method |  |
|  | Library layout/paired-end |  |
|  | N° sequenced reads |  |
| **Raw data** | Reference genome |  |
|  | Annotation version |  |
|  | Mapping method (incl. software, customized settings) |  |
|  | Mapping efficiency |  |
|  | Sequencing saturation |  |
|  | Estimation of ambient RNA |  |
|  | Imputation method and settings |  |
| **Processed data** | N° captured cells/nuclei |  |
|  | N° high quality cells/nuclei |  |
|  | Filter criteria: % mitochondrial reads/cell or nucleus |  |
|  | Filter criteria: % chloroplast reads/cell or nucleus |  |
|  | Filter criteria: Minimum N° UMI/cell or nucleus |  |
|  | N° total detected transcripts |  |
|  | Doublet rate |  |
|  | Replicate comparisons |  |
|  | Batch correction method for merging (incl. reasoning for batch correction) |  |
|  | Additional processing |  |
| **Validation** | Method of automatic annotation of clusters |  |
|  | Method of manual annotation (markers, gene function info) |  |
|  | Verification in planta (e.g. Number of markers used for validation) |  |
| **Data availability** | Analysis scripts & codes (GitHub) |  |
|  | Excel Tables DEG for each cluster |  |
|  | Objects/count matrix in repository (which one, where?) |  |
|  | On-line tool/browser URL |  |
|  | Cell-level metadata table |  |
| **Additional** | additional comments from the authors |  |
